# Improving In Vitro Culture of Human Male Fetal Germ Cells

**DOI:** 10.3390/cells10082033

**Published:** 2021-08-09

**Authors:** Myriam Martin-Inaraja, Monica Ferreira, Jasin Taelman, Cristina Eguizabal, Susana M. Chuva De Sousa Lopes

**Affiliations:** 1 Cell Therapy, Stem Cells and Tissues Group, Basque Centre for Blood Transfusion and Human Tissues, 48960 Galdakao, Spain; myriam.martininaraja@donantesdesangre.eus (M.M.-I.); cristina.eguizabalargaiz@osakidetza.eus (C.E.); 2 Biocruces Bizkaia Health Research Institute, Cell Therapy, Stem Cells and Tissues Group, 48903 Barakaldo, Spain; 3 Department of Anatomy and Embryology, Leiden University Medical Centre, Einthovenweg 20, 2333 ZC Leiden, The Netherlands; monica.ferreira@ua.pt (M.F.); jasin.taelman@gmail.com (J.T.); 4 Ghent-Fertility and Stem Cell Team (G-FaST), Department for Reproductive Medicine, Ghent University Hospital, Corneel Heymanslaan 10, 9000 Ghent, Belgium

**Keywords:** human, male fetal germ cells, extracellular matrix substrate, tissue culture

## Abstract

Male human fetal germ cells (hFGCs) give rise to spermatogonial stem cells (SSCs), which are the adult precursors of the male gametes. Human SSCs are a promising (autologous) source of cells for male fertility preservation; however, in contrast to mouse SSCs, we are still unable to culture them in the long term. Here, we investigated the effect of two different culture media and four substrates (laminin, gelatin, vitronectin and matrigel) in the culture of dissociated second trimester testes, enriched for hFGCs. After 6 days in culture, we quantified the presence of POU5F1 and DDX4 expressing hFGCs. We observed a pronounced difference in hFGC number in different substrates. The combination of gelatin-coated substrate and medium containing GDNF, LIF, FGF2 and EGF resulted in the highest percentage of hFGCs (10% of the total gonadal cells) after 6 days of culture. However, the vitronectin-coated substrate resulted in a comparable percentage of hFGCs regardless of the media used (3.3% of total cells in Zhou-medium and 4.8% of total cells in Shinohara-medium). We provide evidence that not only the choices of culture medium but also choices of the adequate substrate are crucial for optimizing culture protocols for male hFGCs. Optimizing culture conditions in order to improve the expansion of hFGCs will benefit the development of gametogenesis assays in vitro.

## 1. Introduction

Gametes have the extraordinary capacity of transmitting genetic and epigenetic information of an individual to the offspring, thus ensuring the continuity of the species. Fetal germ cells (FGCs), the precursors of male and female gametes, are specified during early mammalian post-implantation development. In humans, FGCs migrate and colonize the gonadal ridges where they undergo sex-specification around 7–10 weeks of gestation (WG), which is equivalent to 5–8 weeks post-fertilization (WPF), by entering either male or female sex-specific pathways [1,2,3,4]. Male FGCs differentiate in the gonadal seminiferous tubules into (mitotically-arrested) prospermatogonia [5]. Starting at puberty, spermatogonial stem cells (SSCs) sustain the male germline and the production of spermatozoa throughout male adult life [6,7,8,9,10]. Although described in rodents [11,12,13] and non-human primates [14,15,16,17], the exact mechanism underlying the transition from prospermatogonia to SSCs is poorly understood in humans. Furthermore, in human postnatal testis, three classes of morphologically different spermatogonia were initially identified (A_dark_, A_pale_ and B) [17,18], but other transcriptionally distinct states within these classes possibly exist, as revealed by recent single-cell analysis [17,19,20,21].

The reconstitution of spermatogenesis, followed by the birth of offspring by means of SSCs-based approaches, has been achieved in mice by in vitro culture of fetal prospermatogonia and postnatal spermatogonia, followed by autologous transplantation [22,23]. Other studies have showed that xenotransplantation of primate testicular tissue into immunodeficient mice resulted in the resumption of spermatogenesis [24,25]. However, transplantation of human testicular tissue into immunodeficient mice has proved less successful since differentiation of human SSCs was limited to spermatocytes [26,27,28]. Other studies have shown the ability of primate SSCs to colonize mouse testis, but those failed to differentiate [24,25,29]. Only autologous transplantation of non-human primate SSCs into busulfan-treated testis supported and improved spermatogenic recovery, resulting in the production of functional sperm capable of fertilizing oocytes and leading to pre-implantation embryo development [6].

Robust protocols exist for in vitro propagation of SSCs isolated from either neonatal or adult testes of numerous species, including rodents [30,31,32,33,34], bovine [35], porcine [36] and marmoset [24], maintaining proliferation capacity and viability. In addition, SSCs seem to require fibroblast feeder layers [30,31,33]; co-culture with testicular somatic cells, such as Sertoli cells and Leydig cells [30,34,37]; or the use of extracellular matrix (ECM) such as laminin [38], gelatin [33], hydrogel [39] or soft-agar [40]. By contrast, a limited number of culture systems have been established for humans [39]. However, by using infant boys, Dong and colleagues succeeded in propagating SSCs enriched after differential plating for five passages [41]. Previous attempts to culture hFGCs from fetal gonads have failed to establish cell lines that maintained germline identity in long-term culture [42,43,44]. Recently, Gell and collaborators [45] reported a co-culture system that maintained hFGC-identity for 10 days in culture. However, mouse embryonic fibroblasts (MEFs) were used as the feeder layer, which is not recommended for future clinical applications.

In the current study, we have used two different culture media that have been previously shown to support the culture of mouse SSCs and that, after further differentiation, were used for fertilization and resulted in viable progeny; we have compared their ability to maintain fetal testicular cells enriched for hFGFs. The Shinohara-medium [46], which contained EGF, FGF2, GDNF, LIF, estrogen and progesterone, supported the 2D expansion on a gelatin-substrate of mouse neonatal SSCs that could be transplanted to infertile recipient males and could restore spermatogenesis and fertility, giving rise to offspring. The Zhou-medium [47], which contained retinoic acid (RA), BMP4 and Activin A (ActA), was used to support the 3D culture of stem cell-differentiated primordial germ cells to the premeiotic SSC-stage. Thereafter, these stem cell-differentiated SSCs were matured to spermatid-like cells that could be used for fertilization, and the generated embryos developed to viable progeny.

Furthermore, we cultured the hFGFs in several different feeder-free substrates: laminin (laminin 521), vitronectin, gelatin (composed of collagens) and Matrigel, which is a hydrogel isolated from mouse sarcoma cells that is composed of a mixture of laminin and collagen. Interestingly, we report the quantification of hFGCs during 6 days of culture, suggesting that vitronectin and gelatin but not Matrigel are suitable feeder-free culture substrates. Overall, our findings provide an optimized feeder-free 2D culture system that sustained male hFGCs and could be used to further investigate the transition from male hFGCs to SSCs.

## 2. Materials and Methods

### 2.1. Ethics for Human Material

The collection and use of human fetal tissue were approved by the Medical Ethical Committee of the Leiden University Medical Centre (P08.087). Human embryos of 16–19.5 WPF from elective abortions were donated for research with signed informed consent. The age of the embryos was determined by obstetric ultrasonography.

### 2.2. Isolation of Male hFGCs

Male gonads were dissected in saline solution (0.9% NaCl, Fresenius Kabi, Bad Homburg, Germany) and either fixed overnight in 4% (*v*/*v*) paraformaldehyde (PFA) for immunohistochemistry or disaggregated by enzymatic digestion, as previously described [30]. Briefly, male gonads were disaggregated with 1 mg/mL collagenase type IV (LS004186, CellSystems, Troisdorf, Germany) and 27 KUnits/mL RNase-free DNAse I (79254, Qiagen, Hilden, Germany) at 37 °C for 105 min (min). The cell suspension was centrifuged (1500 rpm) for 5 min, and the cells were resuspended in Minimum Essential Media (MEM) α medium (22561, ThermoFisher Scientific, Waltham, MA, United States) and plated in 0.1% (*w*/*v*) gelatin (G7041, Merck, Kenilworth, NJ, United States)-coated petri dish (10 cm diameter) (Corning, Corning, NY, United States) at 37 °C for 30 min in order to reduce the amount of gonadal somatic cells by differential cell adhesion selection. After incubation, the cell suspension enriched for hFGCs (non-adherent fraction) was collected for fluorescence-activated cell sorting (FACS) analysis or for further culture.

### 2.3. FACS Analysis of Male hFGCs

In order to quantify the enrichment of male hFGCs obtained in the non-adherent fraction, we determined the percentage of hFGCs after the differential adhesion procedure by using surface markers THY1 and ITGA6, which are both established markers for identifying hFGCs and Sertoli cells [48,49,50,51], in the dissociated male gonads (before plating) and in the adherent fraction and non-adherent fraction by flow cytometry analysis. Cells were washed with FACS-buffer consisting of 10% fetal bovine serum (FBS) in phosphate-buffered saline buffer (PBS) and incubated for 30 min at 4 °C with conjugated antibodies diluted in FACS-buffer (Table S1). Cell were analyzed in a FACS Canto II (BD Biosciences, Franklin Lakes, NJ, United States). Events numbering 25,000 were acquired for analyses. Populations were analyzed by using FlowJo v.X.0.7 (TreeStar Inc, Ashland, OR, United States).

### 2.4. Culture of Male hFGCs

The non-adherent fraction of the male gonadal cells (*n* = 5 different donors) was gently collected, centrifuged and resuspended in the following: (1) Shinohara-medium (StemPro-34 supplemented with 30 ng/mL β-estradiol (E2758-250MG, Sigma Aldrich, St. Louis, MO, United States); 60 ng/mL progesterone (P8783-1G, Sigma Aldrich); 20 ng/mL human epidermal growth factor (hEGF) (130-093-825, Miltenyi Biotec, Leiden The Netherlands); 10 ng/mL human basic fibroblast growth factor (FGF2) (130-104-924, Miltenyi Biotec); 10 ng/mL human glial cell line-derived neurotrophic factor (hGDNF) (130-096-291, Miltenyi Biotec); and 10 ng/mL human leukemia inhibitor factor (hLIF) (130-108-157, Miltenyi Biotec), as described previously [46]) or (2) adapted Zhou-medium (αMEM supplemented with 10% KnockOut Serum Replacement (KSR) (Life Technologies, Carlsbad, CA, United States); 20 ng/mL BMP4 (314-BP-010/CF, R&D Systems, Minneapolis, MN, United States); 100 ng/mL Activin A (338-AC-010/CF, R&D Systems); and 1 μM retinoic acid (R2625, Merck) [47]). The non-adherent fraction of the male gonads was plated on 6 individual wells of 24-well plates (50,000 cells/well), each well containing a glass coverslip (13 mm diameter) coated with the following: 5 µg/mL laminin (LN 521, BioLamina, Sundbyberg, Sweden); 0.1% (*w*/*v*) gelatin (G7041, Merck); 3 µg/mL human recombinant vitronectin (rhVTN) (A14700, Life Technologies,); or 1% (*w*/*v*) matrigel (354230, BD Bioscience). The media were refreshed every 2 days, and hFGCs were cultured for 3 or 6 days in 5% CO_2_ on air at 37 °C.

### 2.5. Immunofluorescence and Histology

Male gonads (16-19.5 WPF) (*n* = 3 different donors) were fixed in 4% PFA overnight at 4 °C, dehydrated in a series of ethanol and embedded in paraffin by using standard procedures. Paraffin sections (5 μm thickness) were deparaffinized in xylene, followed by a series of ethanol dilutions and distilled water. Antigen retrieval was performed by heating the sections (98 °C) submerged in 10 mM sodium citrate buffer (pH 6.0) for 15 min using a microwave (TissueWave 2, ThermoFisher Scientific, Waltham, MA, United States) and allowed to cool down. After blocking in 3% bovine serum albumin (BSA)/PBS at room temperature (RT) for 1 h, the sections were incubated overnight with the primary antibodies (Table S1), followed by incubation with the secondary antibodies at RT for 2 h, counterstained with DAPI and mounted with ProLongTM Gold antifade reagent (P36930, ThermoFisher Scientific). For negative controls, the staining procedures were as described above, except that the primary antibodies were omitted.

The non-adherent fractions of the male gonads (*n* = 5 different donors), which were seeded on microscope coverslips (50,000 cells/well of 24-well plate), were processed for immunofluorescence after 3 days (D3) and 6 days (D6) in culture. Briefly, cells were washed with PBS and immediately fixed with 4% PFA at RT for 20 min. After fixation, the cells were permeabilized with 0.1% (*v*/*v*) Triton X-100 in PBS for 8 min and blocked with 3% (*w*/*v*) BSA (Sigma-Aldrich) in PBS for 1 h. Following 3 washing steps with PBS, the cells were incubated for at RT 2 h with the primary antibodies (Table S1). Thereafter, cells were incubated at RT for 1 h with secondary antibodies (Table S1) in 3% BSA in PBS. Following 5 min of washing with PBS, the cells were counterstained with DAPI and mounted on slides with ProLongTM Gold.

### 2.6. Imaging, Quantification and Statistical Analysis

Bright field images were obtained using a DMi8 microscope (Leica, Wetzlar, Germany) with a 10×/0.4 objective. Immunofluorescence images were obtained with a slide scanner microscope Axio Scan Z1 (Zeiss, Oberkochen, Germany) with a 20×/0.95 objective and an ApoTome.2 (Zeiss) with a 63×/1.4 objective. The quantification of hFGCs in the male gonads (*n* = 3 different biological donors) was performed by counting the hFGCs present in 6–10 different images (90,000 μm^2^) and 2 images per paraffin section of each biological donor. The quantification of cultured hFGCs (*n* = 5 different biological donors) was performed by counting cells in 10 different images (90,000 μm^2^), covering different regions of each coverslip (13 mm diameter) and summing them. The total number of cells counted per biological replicate is shown in Table S2. Image analysis and cell quantification were conducted using the microscope software ZEN 3.1 (Zeiss, Oberkochen, Germany) and ImageJ [52]. GraphPad Prism 8.4.2 software (GraphPad, San Diego, CA, United States) was used to generate graphs and statistical analysis was performed by using the Student’s *t*-test and the one-way ANOVA test (*, *p*-value < 0.05; **, *p*-value < 0.01; ***, *p*-value < 0.001; ns, not significant) as indicated. Data are shown as mean ± standard error of the mean (SEM) or mean ± standard deviation (SD), as indicated.

### 2.7. Analysis of Single-Cell Transcriptomics Data

Single cell RNA sequencing (RNASeq) data from human fetal male gonads (previously published by Li and colleagues [3] and available online) were analyzed in R (v4.0.2). Unique Molecular Identifier (UMI) count data (GSE86146) and metadata were used as provided. From the dataset available, we selected male cells corresponding only to the second trimester (19–25 WPF; *n* = 5 different biological donors), comparable to the fetal testes used for culture. Subsequently, we removed cells with <2,000 genes and >100,000 counts. Next, transcripts per million (TPM) values were calculated as counts/(total counts per sample) × 10^6^. TPM values were used to create the Seurat object, including genes with TPM >1 in at least 10 cells. TPM values were normalized with log_e_-transformation within the Seurat workflow. By using the FindVariableFeatures and vst as the selection methods, the 2,000 most variable genes were calculated, scaled and centered in the data and used as input for further analysis. Principal component analysis (PCA) was performed with RunPCA. Next, tSNE plots were generated by using FindNeighbours, FindClusters and RunTSNE with Dimensions = 18 and Resolution = 0.8, visualized by Cluster ID and age (WPF). Violin plots were generated using VlnPlot for markers of interest. tSNE plots visualizing the expression of genes of interest were generated with FeaturePlot.

## 3. Results

### 3.1. Second Trimester Male Fetal Gonads Contained Populations of hFGCs Differing in the Expression of POU5F1 and DDX4

**Figure 1:**
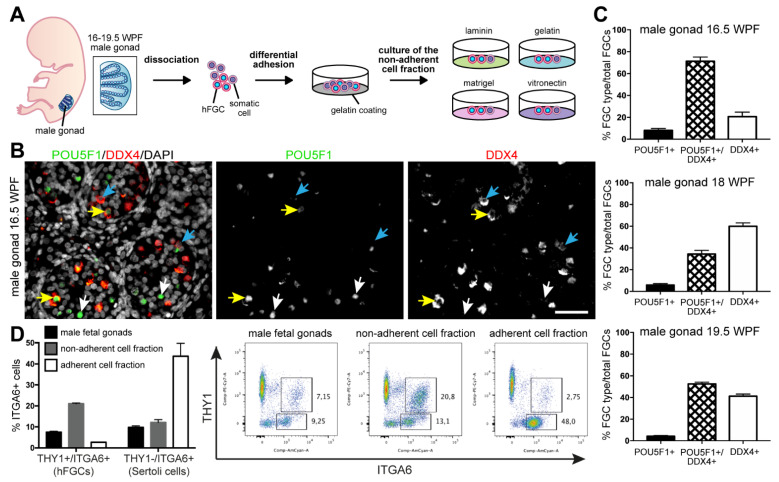
Characterization of POU5F1 and DDX4 expression in hFGCs in human fetal testes. (**A**) Experimental design used in this study. (**B**) Immunofluorescence for (nuclear) POU5F1 and (cytoplasmic) DDX4 in male gonads of 16.5 WPF. White arrows show POU5F1+ hFGCs, yellow arrows show double POU5F1+/DDX4+ hFGCs and blue arrows show DDX4+ hFGCs. Scale bar is 50 µm. (**C**) Percentage of POU5F1+, double POU5F1+/DDX4+ and DDX4+ hFGCs in male gonads of 16.5, 18 and 19.5 WPF (*n* = 3 different donors) (mean ± SEM). (**D**) hFGC enrichment analysis. Quantification of the percentage of THY1+/ITGA6+ and THY1-/ITGA6+ cells in the male fetal gonads of 19 WFP in the adherent and in the non-adherent fraction (mean ± SEM) and representative FACS dot-plots.

In the current study, we investigated whether hFGCs from fetal male gonads of the second trimester could propagate in vitro in the presence of residual Sertoli cells and Leydig cells when cultured in several different types of ECM (gelatin, laminin, vitronectin and matrigel) (Figure 1A). We started by quantifying the percentage of male hFGCs expressing POU5F1 and DDX4 in histological sections of male gonads from 16.5 to 19.5 WPF (Figure 1B,C).

Three populations of male hFGCs were observed on the basis of the relative co-expression of POU5F1 and DDX4 (Figure 1B; Figure S1A). After quantification of the different population of hFGCs, we showed that only about 10% of the hFGCs consisted of DDX4-POU5F1+, whereas the rest expressed DDX4+ (Figure 1C). Next, we performed differential adhesion (30 min on gelatin-coated plates) to enrich the population of male hFGCs. This is the time interval and substrate used to deplete pluripotent stem cells from the MEF feeder layer. As the human fetal testes have comparable developmental age and hFGCs exhibit characteristics in common with pluripotent stem cells, we reasoned that preplating for 30 min would be sufficient for the somatic cells in the male testes to adhere to the gelatin-coated plate. By using FACS, we compared the percentage of male hFGCs known to express the surface markers THY1 (also known as CD90) and ITGA6 [48,49,50,51] before and after enrichment and observed an 3 fold enrichment (average of 7% in the gonad compared with 23% in the non-adherent fraction) (Figure 1D; Figure S1B). Note that after 30 min, the percentage of hFGCs that remained in the adherent fraction was on average 3%, suggesting that longer incubation time, such as overnight incubation as used by Kanatsu-Shinohara and colleagues [53], may not be necessary. After 30 min, the adherent fraction consisted of a 4–5 fold enrichment in THY1-ITGA6+ Sertoli cells [49,51] (average of 10% in the gonad compared with 45% in the adherent fraction) (Figure 1D).

### 3.2. The Substrates Gelatin and Vitronectin Sustained In Vitro Culture of hFGCs

**Figure 2:**
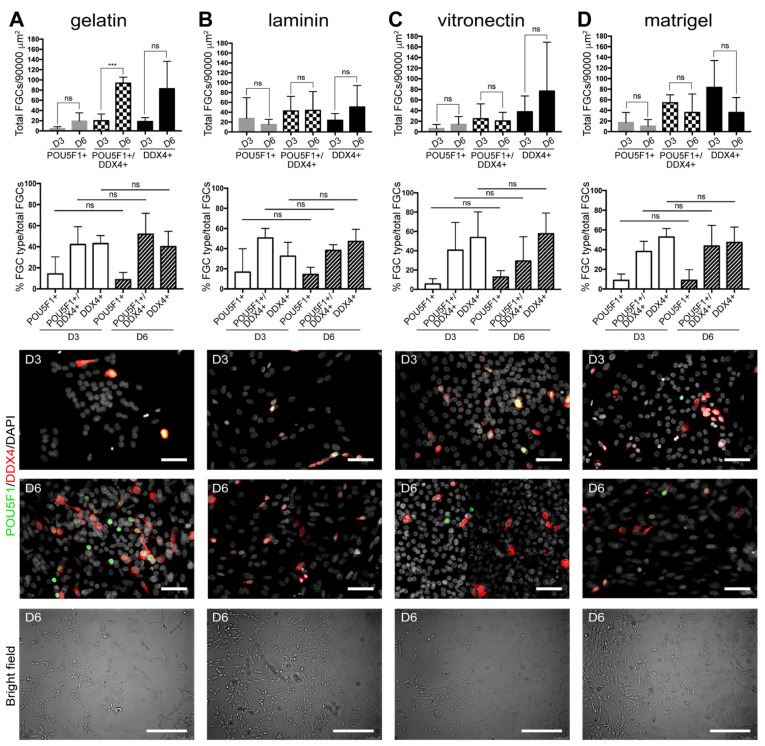
Quantification of hFGCs in Shinohara-medium and different substrates. Quantification of the total number of hFGCs (*n* = 3 different donors) per 90,000 μm^2^ and the percentage of different hFGC types (POU5F1+, POU5F1+DDX4+ and DDX4+ cells) grown for 3 and 6 days in gelatin-coated substrate (**A**), laminin-coated substrate (**B**), vitronectin-coated substrate (**C**) and matrigel-coated substrate (**D**). All data are represented as mean ± SEM. Statistical analysis was performed by using the *t*-test (***, p-value < 0.001; ns, not significant). For immunofluorescence, nuclei were counterstained with DAPI (white). Scale bars: 50 µm in immunofluorescence images and 300 µm in bright field images.

We cultured an enriched population of male hFGCs (about 20% of the gonadal cells) in the presence of Sertoli and Leydig cells for 3 days (D3) and 6 days (D6) in Shinohara-medium containing EGF, FGF2, GDNF, LIF, estrogen and progesterone on four different ECM substrates (gelatin, laminin, vitronectin and matrigel) (Figure 2; Table 1; Figure S1C). The total number of cells (DAPI+) doubled between D3 and D6 in all conditions, except in matrigel (Table 1). As a readout, we quantified and compared the total number and percentage of hFGCs expressing DDX4 and POU5F1. The adherent hFGCs showed typical morphology and an increasing trend in number between D3 and D6 when grown in gelatin (Figure 2A) and vitronectin (Figure 2C) but not on laminin (Figure 2B), and a decreasing trend in matrigel was observed (Figure 2D). Between D3 and D6, we observed a statistically significant increase in the total number of POU5F1+/DDX4+ hFGCs (Figure 2A), accompanied by a statistically significant decrease in the percentage of (POU5F1-/DDX4-) somatic gonadal cells (Table 1; Figure S1C) in Shinohara-medium on gelatin. Moreover, about 10% of the total (DAPI+) cells were hFGCs on D6 when grown in gelatin, whereas only 5% of the total (DAPI+) cells were hFGCs on the other substrates (Table 1).

**Figure 3:**
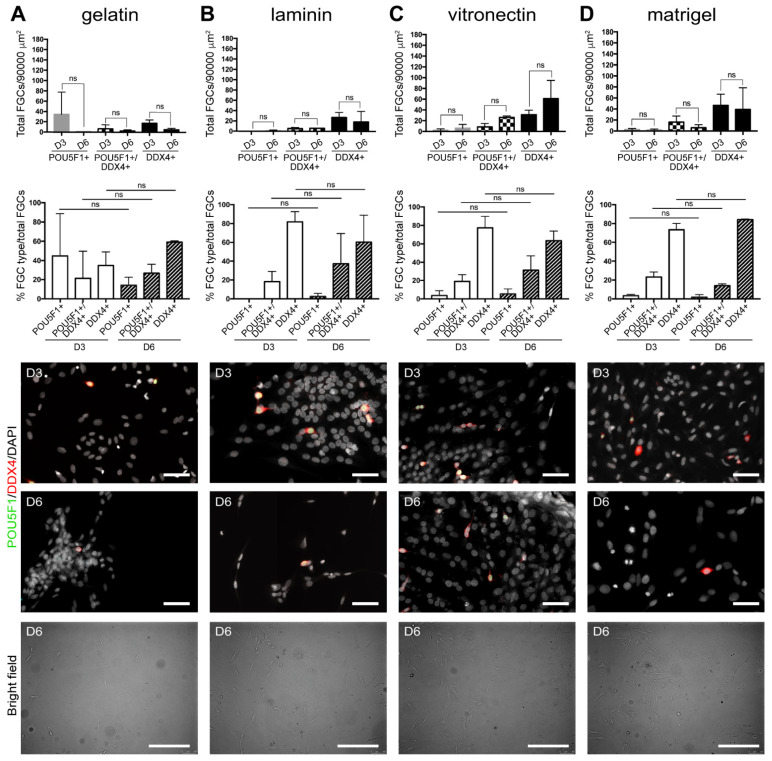
Quantification of hFGCs in the adapted Zhou-medium and different substrates. Quantification of the total number of hFGCs (*n* = 2 different donors) per 90,000 μm^2^ and the percentage of different hFGC types (POU5F1+, POU5F1+DDX4+ and DDX4+ cells) grown for 3 and 6 days in gelatin-coated substrate (**A**), laminin-coated substrate (**B**), vitronectin-coated substrate (**C**) and matrigel-coated substrate (**D**). All data are represented as mean ± SEM. Statistical analysis was performed by using the *t*-test (ns, not significant). For immunofluorescence, nuclei were counterstained with DAPI (white). Scale bars: 50 µm in immunofluorescence images and 300 µm in bright field images.

**Figure 4:**
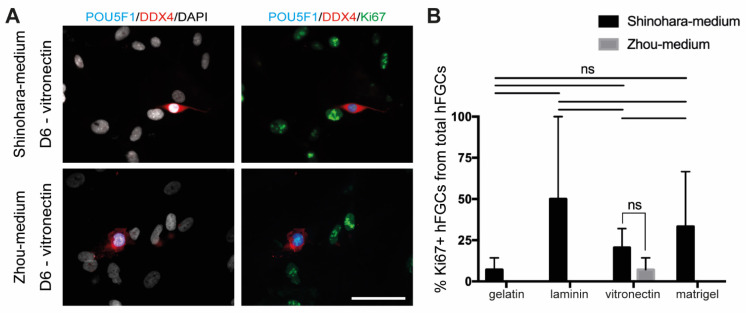
Germ cell proliferation analysis. (**A**) Immunofluorescence for Ki67, DDX4 and POU5F1 in hFGCs cultured for 6 days in Shinohara-medium or Zhou-medium in vitronectin-coated substrate. In the left panels, DAPI is shown together with DDX4 and POU5F1; in the right panels, the same field is shown for Ki67, DDX4 and POU5F1. Scale bar: 50 μm. (**B**) Quantification of the percentage of Ki67+ hFGCs (*n* = 2 different donors) from total hFGCs (POU5F1+/DDX4-, POU5F1+/DDX4+ and POU5F1-/DDX4+) cultured for 6 days in Shinohara-medium or Zhou-medium on each of the coating-substrates used (gelatin, laminin, vitronectin and matrigel) (mean ± SEM). Statistical analysis was performed by using the one-way ANOVA between the multiple substrates on Shinohara-medium and by using the t-test between the two media in vitronectin (ns, not significant).

When hFGCs from dissociated 16–19.5 WPF male fetal gonads were cultured for D6 in adapted Zhou-medium containing RA, BMP4 and ActA but not BMP2 and BMP7 (Figure 3; Table 1; Figure S1D), we observed a decreasing trend in the number of hFGFs grown in gelatin (Figure 3A), laminin (Figure 3B), vitronectin (Figure 3C) and matrigel (Figure 3D). After culture in the adapted Zhou-medium, the total number of cells (DAPI+) doubled between D3 and D6 in all conditions, except for gelatin (Table 1). When grown on vitronectin for 6 days, the hFGFs represented on average 3.3% of the total (DAPI+) cells compared to about 2% on the other substrates. We have quantified the presence of proliferative (Ki67+) hFGCs in the total population of hFGCs (POU5F1+/DDX4-, POU5F1+/DDX4+ and POU5F1-/DDX4+) after 6 days of culture in the adapted Zhou-medium and observed that vitronectin was the only substrate that supported hFGC proliferation (Figure 4). This contrasted with culture in Shinohara-medium that showed some degree of proliferative (Ki67+) hFGCs in the total population of hFGCs (POU5F1+/DDX4-, POU5F1+/DDX4+ and POU5F1-/DDX4+) on all substrates (Figure 4). We also observed that the majority of (POU5F1-DDX4-) somatic gonadal cells after 6 days of culture showed positivity for Ki67 (Figure 4A).

In conclusion, vitronectin is an adequate substrate to maintain hFGFs at least during the first 6 days of culture in both media tested; however, the highest percentage of hFGCs (about 10%) was obtained when hFGFs were cultured on gelatin in Shinohara-medium.

### 3.3. Gene Expression Single Cell RNA Sequencing Analysis

**Figure 5:**
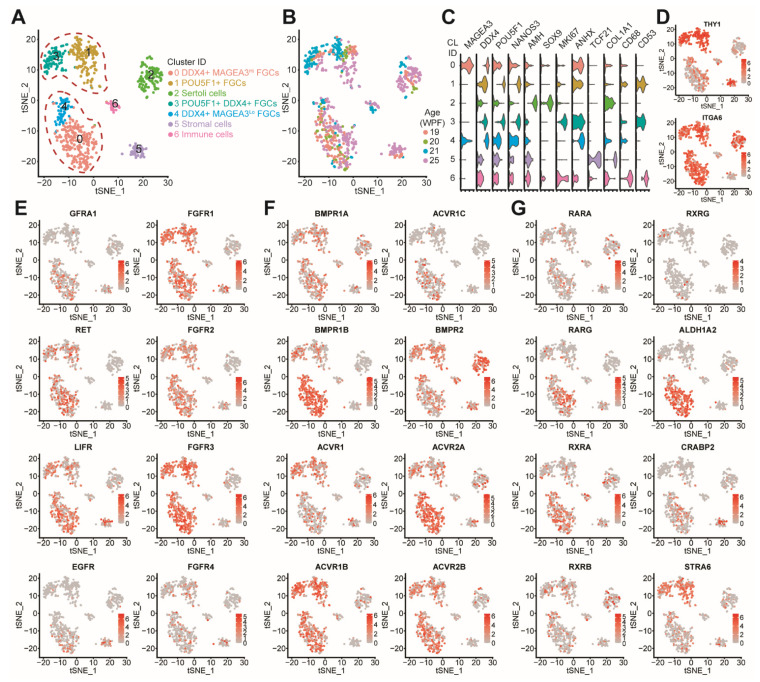
Gene expression analysis of single cell RNASeq data of male fetal gonads. (**A**) tSNE plot of single cell data from Li et al. (2017) (*n* = 5 different donors). A total of 7 clusters (cluster 0–6) were identified. (**B**) t-SNE plot colored by age in weeks post fertilization (WPF). (**C**) Violin plots showing expression of representative genes for each cluster. (**D**) tSNE feature plots showing the expression of THY1 and ITGA6. (**E**) tSNE feature plots showing the expression of specific receptors associated with signaling pathways activated by the Shinohara-medium. (**F**) tSNE feature plots showing the expression of specific receptors associated with the BMP-ActA signaling pathway activated by the adapted Zhou-medium. (**G**) tSNE feature plots showing the expression of specific receptors associated with the RA signaling pathway activated by the adapted Zhou-medium.

In order to explain the differences observed in the number of hFGCs in the two media tested (adapted Zhou-medium and Shinohara-medium), we have analyzed the expression of the receptors needed to activate the signaling pathways associated with the different growth factors present in the two different media used in male hFGCs. In order to perform this, we made use of an online-available single-cell sequencing dataset (Smart-Seq2) from human fetal gonads [3]. From that database, we have extracted the data of the gonadal male cells from 19–25 WPF, which are comparable to the fetal testes analyzed here, and used a Seurat-workflow [54] to cluster the male gonadal cells and identified seven clusters (clusters 0–6) (Figure 5A), with a relative contribution from all ages (Figure 5B). We identified three clusters (CL) of gonadal somatic cells: CL6 corresponded to CD68+CD53+ immune cells, CL5 to TCF21+COL1A1+ stromal cells and CL2 to AMH+SOX9+ Sertoli cells. We also identified four CL of hFGCs: CL1 corresponded to POU5F1+NANOS3+ hFGCs, CL3 to POU5F1+NANOS3+DDX4+ hFGCs, CL4 to DDX4+/MAGEA3^Lo^ hFGCs and CL0 to DDX4+/MAGEA3^Hi^ hFGCs (Figure 5C). Next, we confirmed that hFGCs expressed both THY1 and ITGA6 (Figure 5D).

The Shinohara-medium contained EGF, FGF2, GDNF and LIF. Interestingly, EGF, FGF2 and GDNF are ligands to receptor tyrosine kinases EGFR, FGFR (FGFR1-4) and GFRA1/RET, respectively, and all resulted in the activation of MAPK and PI3K signaling [55]. LIF binds to the LIFR, which can subsequently activate JAK/STAT, MAPK and PI3K signaling [56]. Interestingly, GFRA1 and EGFR were expressed by only a few DDX4+ hFGCs in CL0, whereas RET, LIFR and FGFR1-3 were expressed by all subtypes of hFGCs (Figure 5E). Together, although the Shinohara-medium seemed to support hFGCs culture, the addition of EGF may not be important in order to maintain hFGCs as they will not be able to respond to that signaling pathway.

The adapted Zhou-medium contained RA, BMP4 and ActA. BMP4 and ActA are both ligands to the TGFβ superfamily. BMP4 binds to BMP type II receptor (BMPR2) and several BMP type I receptors (BMPR1A (or Alk3), BMPR1B (or Alk6) and ACVR1 (or Alk2)), and ActA binds to Activin type II receptors (ACVR2A and ACVR2B) and Activin type I receptors (ACVR1B (or Alk4) and ACVR1C (or Alk7)) [57]. We investigated the expression of the BMP type I receptors and Activin type I receptors and observed that DDX4+ hFGCs in CL0 expressed high levels of BMPR1B, whereas ACVR1B was expressed by all types of hFGCs (Figure 5F). All type II receptors seemed to be expressed in hFGCs (Figure 5F). RA signals via nuclear retinoic acid receptors (RARs) and retinoic X receptors (RXRs) [58]; however, only some DDX4+ hFGCs in CL0 seemed to express RA receptors (Figure 5G), suggesting that DDX4+ hFGCs are able to activate RA signaling. Interestingly, DDX4+ hFGCs in CL0 also expressed high levels of ALDH1A2, which is the enzyme that produces RA from retinaldehyde.

In the future, it will be interesting to combine the addition of FGF2, GDNF and LIF with BMP4, ActA and RA to hFGCs and to evaluate the effect on proliferation as well as a possible transition to mitotically-active SSCs.

## 4. Discussion

The autologous transplantation of SSCs holds great therapeutical potential once applied to patients. However, none of the described techniques have reached the preclinical or clinical stage due to the existence of imminent risks, namely cancer relapse due to neoplastic cell contamination from non-sorted testicular cell suspensions [20,26]. Moreover, human testicular biopsies do not contain sufficient SSCs for recolonization of the seminiferous tubules after transplantation. Hence, understanding how to generate FGCs-derived SSCs in vitro constitutes a viable strategy to further investigate how human SSCs can be propagated in order to obtain an adequate number of cells for successful transplantation into human patients. In addition, it would stimulate research on the production and maturation of male gametes in vitro. Long term maintenance of human SSCs is essential for their potential clinical applications and for a future strategy to restore the fertility in humans.

In the present study, we have compared two culture media in order to expand and maintain 16–19.5 WPF male hFGCs. The Shinohara-medium was described previously for the maintenance and proliferation of mouse SSCs in feeder-free condition [46] and for human SSCs [59,60]. This medium provides key growth factors that induce germ cell propagation and survival. Several differences were found between our four different feeder-free culture conditions with the Shinohara-medium. In previous studies, gelatin has been shown to promote cell adhesion, proliferation and migration of human pluripotent stem cells (hiPSCs) and mouse SSCs in vitro [61,62,63]. Other studies show that gelatin-coated plates also induce the proliferation of male bovine SSCs [64] and human male germ stem cell-like cells [65]. The ECM protein vitronectin binds to integrin receptors, allowing cell survival and proliferation [66]. In addition, vitronectin has been used to support human embryonic stem cells (hESCs) [67] and hiPSCs propagation in vitro [68]. Laminin and matrigel were less successful in the maintenance of hFGCs in culture. Matrigel is composed of laminin, collagen IV, heparin sulfate and growth factors. Laminin and collagen IV are secreted by Sertoli cells [69,70] and may be more relevant for maintaining this cell type.

Our results show that male hFGCs showed the highest relative number in culture when cultured with Shinohara-medium, meaning that the culture needs growth factors EGF, FGF2, GDNF and LIF for the survival and self-renewal of germ cells as described previously for hSSCs [59,71] and mSSCs [46,72,73]. As Kitajima and Niwa described, FGF2 is effective for stabilizing ESCs self-renewal [63]. In addition, GDNF and LIF has also been used for the in vitro expansion of mouse SSCs [62], but the addition of EGF due to the lack of the receptor EGFR may be dispensable in order to maintain hFGCs in culture.

Regarding the use of the Zhou-medium, the role of BMP signaling was been previously studied in male hFGCs from seven WPF [2]. There, it was shown that male hFGCs from seven WPF responded to BMP signaling inhibition after culture for 24 h by remodeling chromatin accessibility to promoters associated with the WNT signaling pathway. However, this was not investigated in hFGCs of the second trimester. Moreover, previous studies that have cultured (pluripotent) cells in the presence of ActA, RA and BMPs have reported the formation of PGC-like cells (PGCLCs) in vitro [74,75], as well as the promotion of gametogenesis in vitro [47]. Studies on the differentiation of human PGCLCs from hiPSCs or hESCs have also reported the need for the addition of BMPs and ActA to the differentiation medium [8,45,76,77,78,79]. However, as other studies reported that BMP4 was sufficient to promote PGCLC differentiation [80], we have omitted the use of BMP2 and BMP7 originally present in the Zhou-medium. We cannot currently exclude that supplementation with BMP2 and BMP7 would be beneficial for the culture of male hFGCs. Studies in mice have showed that RA was important to induce male germline differentiation [81]. Interestingly, although hFGCs contain the relevant receptors, the numbers of hFGCs showed a declining trend in vitro when cultured with adapted Zhou-medium (containing ActA, RA and BMP4 but lacking BMP2 and BMP7), except in vitronectin. This suggests that not only the choices of culture media but also the choices of substrate are crucial and should be further investigated in order to optimize culture conditions of hFGCs.

In conclusion, this work makes a contribution by exploring the differences in feeder-free culture systems regarding media and substrate composition in order to maintain male hFGCs in culture for 6 days. In our study, the male hFGCs after 6 days in culture may have either entered senescence or become overgrown by (proliferating) somatic gonadal cells. We suggest that long-term culture may require a subsequent step of either differential adhesion in order to further enrich hFGCs or, alternatively, require FACS sorting as recently described in order to expand human PGCLCs [82]. Although our study used a limited number of different biological donors, our results demonstrate the impact of using different substrates to culture male hFGCs, suggesting that, in addition to the factors present in the medium, the chosen coating-substrates also influence the success of the culture protocol. The relative number of hFGCs was the highest (mean of 10% hFGCs of total cells) in the Shinohara-medium (containing EGF, FGF2, GDNF and LIF) on a gelatin coated-substrate, with a statistically significant increase in the number of POU5F1+/DDX4+ hFGCs accompanied with a statistically significant decrease in the number of POU5F1-/DDX4- gonadal somatic cells between D3 and D6. Finally, we report a comparable percentage of hFGCs on vitronectin on D6 regardless of the media used (mean of 3.3% hFGCs of total cells in Zhou-medium versus a mean of 4.8% hFGCs of total cells in Shinohara-medium). Our data contributes to deciphering the optimal conditions for investigating hFGC proliferation and differentiation, which are important for developing gametogenesis assays in vitro for future male infertility treatment.

## Figures and Tables

**Table 1:** Quantification of male hFGCs after culture in the different conditions.

	*n*	Mean DAPI+ Cells ± SD	Mean DDX4+ Cells ± SD (% Per DAPI+ ± SD)	Mean DDX4+/POU5F1+ Cells ± SD (% Per DAPI+ ± SD)	Mean POU5F1+ Cells ± SD (% Per DAPI+ ± SD)	Mean SOMA Cells ± SD (% Per DAPI+ ± SD)
		Shinohara-Medium, with EGF, FGF2, GDNF, LIF, Estrogen and Progesterone				
		D3				
Gelatin	3	792.0 ± 388.8	18.3 ± 7.6 (2.3%± 0.7)	20.0 ± 13.0 (2.5% ± 1.0)	4.3 ± 3.8 (0.6% ± 0.9)	749.3 ± 372.1 (94.6% ± 0.8)
Laminin	3	1259.3 ± 915.3	23.7 ± 13.7 (1.9% ± 1.4)	42.3 ± 29.7 (3.4% ± 4.0)	27.3 ± 42.3 (2.2% ± 5.5)	1166.0 ± 920.8 (92.6%±11.0)
Matrigel	3	2252.3 ± 356.6	83.3 ± 50.5 (3.7% ± 3.1)	54.3 ± 15.0 (2.4% ± 1.1)	17.0 ± 19.3 (0.8% ± 1.1)	2097.7 ± 437.9 (93.1% ± 5.3)
Vitronectin	3	1092.7 ± 827.7	37.7 ± 30.0 (3.4% ± 2.2)	24.7 ± 28.1 (2.3% ± 1.1)	6.0 ± 7.9 (0.6% ± 0.4)	1024.3 ± 766.5 (93.8% ± 1.8)
		D6				
Gelatin	3	2028.3 ± 767.9	82.7 ± 53.8 (4.1% ± 1.1)	9.,7 ± 11.7 (4.6% ± 2.2)	19.3 ± 15.9 (1.0% ± 0.7)	1832.7 ± 709.2 (90.4% ± 0.8)
Laminin	3	2931.0 ± 1973.9	50.7 ± 43.6 (1.7% ± 2.2)	43.7 ± 38.4 (1.5% ± 1.9)	15.0 ± 10.5 (0.5% ± 0.6)	2821.7 ± 1994.9 (96.3%±4.6)
Matrigel	3	1405.0 ± 694.3	36.0 ± 28.1 (2.6% ± 1.1)	36.0 ± 35.1 (2.6% ± 2.7)	10.3 ± 12.3 (0.7% ± 0.6)	1322.7 ± 650.2 (94.1% ± 3.4)
Vitronectin	3	2331.0 ± 1061.9	76.7 ± 92.2 (3.3% ± 2.2)	20.7 ± 15.9 (0.9% ± 0.9)	14.0 ± 14.8 (0.6% ± 0.3)	2219.7 ± 960.4 (95.2% ± 2.1)
		Zhou-Medium (Adapted), with RA, BMP4 and ActA				
		D3				
Gelatin	2	1060.5 ± 548.0	17.5 ± 6.4 (1.7% ± 1.7)	6.5 ± 7.8 (0.6% ± 0.5)	34.5 ± 43.1 (3.3% ± 6.6)	1002.0 ± 589.7 (94.5% ± 7.8)
Laminin	2	928.5 ± 593.3	27.0 ± 9.9 (2.9% ± 1.0)	5.5 ± 2.1 (0.6% ± 0.8)	0.0 ± 0.0 (0.0% ± 0.0)	896.0 ± 585.5 (96.5% ± 1.8)
Matrigel	2	1148.5 ± 7.8	46.5 ± 2.,5 (4.1% ± 1.8)	16.0 ± 11.3 (1.4% ± 1.0)	2.5 ± 2.1 (0.2% ± 0.2)	1083.5 ± 41.7 (94.3% ± 3.0)
Vitronectin	2	1387.0 ± 56.6	31.0 ± 8.5 (2.2% ± 0.5)	8.5 ± 6.4 (0.6% ± 0.4)	2.0 ± 2.8 (0.1% ± 0.2)	1345.5 ± 38.9 (97.0% ± 1.2)
		D6				
Gelatin	2	853.0 ± 540.2	5.0 ± 2.8 (0.6% ± 0.1)	2.5 ± 2.1 (0.3% ± 0.1)	1.0 ± 0.0 (0.1% ± 0.1)	844.5 ± 535.3 (99.0% ± 0.1)
Laminin	2	1833.5 ± 1826.5	18.5 ± 20.5 (1.0% ± 0.2)	6.0 ± 0.0 (0.3% ± 0.7)	1.0 ± 1.4 (0.1% ± 0.0)	1808.0 ± 1804.5 (98.6%±0.4)
Matrigel	2	1955.5 ± 170.4	39.0 ± 39.6 (2.0% ± 1.9)	6.0 ± 5.7 (0.3% ± 0.3)	1.5 ± 2.1 (0.1% ± 0.1)	1909.0 ± 123.0 (97.6% ± 2.2)
Vitronectin	2	2819.0 ± 582.7	61.0 ± 33.9 (2.2% ± 0.8)	26.0 ± 2.8 (0.9% ± 0.3)	6.0 ± 7.1 (0.2% ± 0.2)	2726.0 ± 544.5 (96.7% ± 0.7)

## Data Availability

The dataset used in this publication is publicly available from NCBI Gene Expression Omnibus (GEO) with accession number GSE86146.

## Supplementary Materials

The following are available online at https://www.mdpi.com/article/10.3390/cells10082033/s1, Figure S1: Negative controls, FACS gating strategy and visualization associated with Table 1. Table S1: List of antibodies used in this study. Table S2: Total number of DAPI+ cells counted in each biological donor (associated with Table 1).
