# Supplementary material for: Improving In Vitro Culture of Human Male Fetal Germ Cells
Source: Cells. 2021 Aug 9;10(8):2033. doi: 10.3390/cells10082033 (PMC8393746; doi:10.3390/cells10082033)
Supplement: Supplementary file 1 [file cells-10-02033-s001.zip › cells-1225466-suppl.pdf]

cells MDPI

Supplementary Materials:

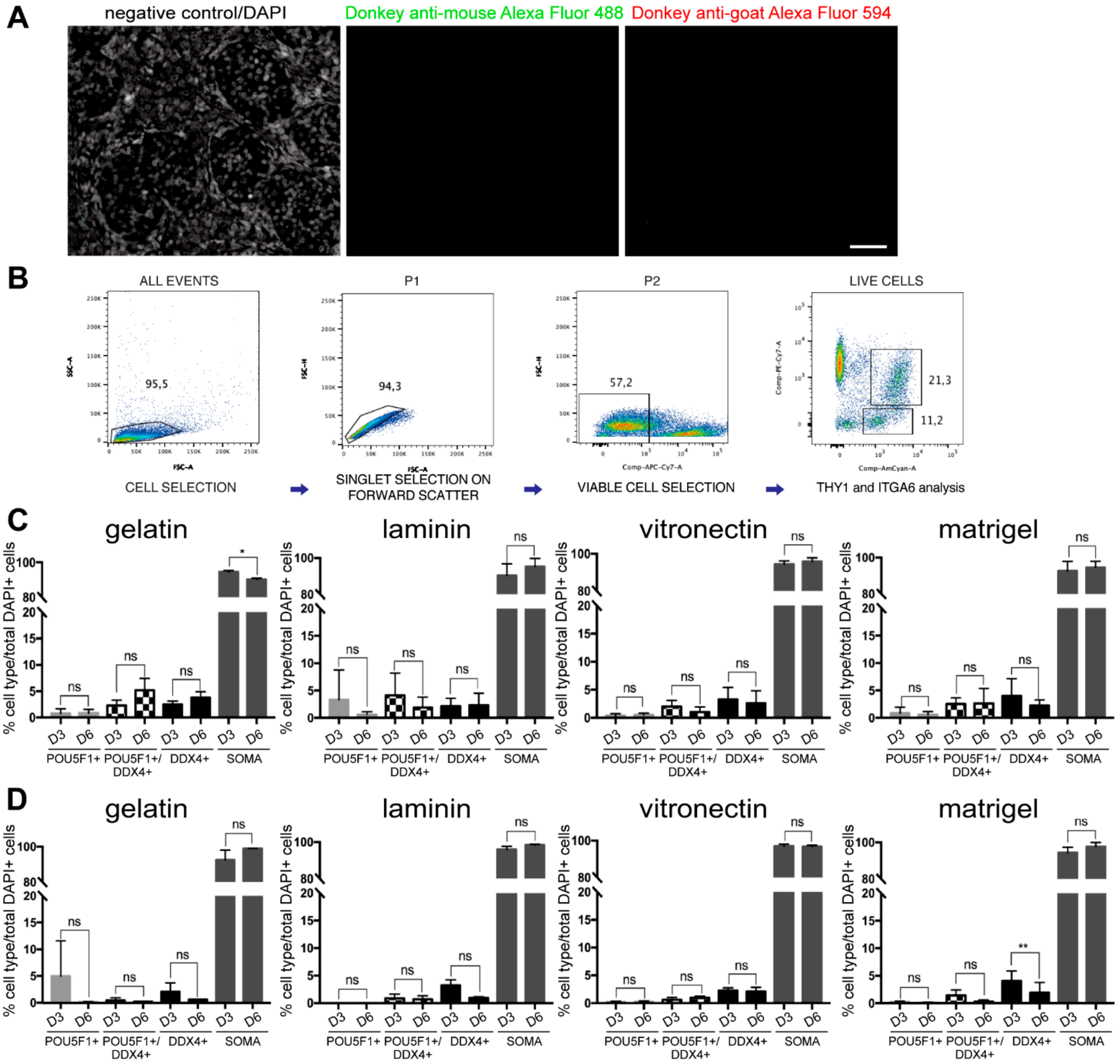

Supplementary Figure S1. Negative controls, FACS gating strategy and visualization associated with Table 1. A) Negative control for the immunofluorescence using secondary antibodies only in male gonads of 16.5 WPF. Scale bar: 50 µm. **B)** FACS gating strategy showing representative dot-plots for the gates used to select population 1 (P1), singlets (P2), viable cells and finally the expression analysis of THY1 and ITGA6. **C)** Percentage hFGCs and gonadal somatic cells per DAPI+ cells (N=3 donors) after culture for D3 and D6 in Shinohara-medium on the different substrates (mean ± SD). **D)** Percentage of hFGCs and gonadal somatic cells cells per DAPI+ cells (N=2 donors) after culture for D3 and D6 in Zhou-medium on the different substrates (mean ± SD). Statistical analysis was performed using the t-test. The graphs are a visualization of the mean percentages shown in Table 1.

Table S1. List of antibodies used in this study.

Antibody	Company (Cat. no.)	Dilution
goat anti-DDX4 (VASA)	R&D Systems (AF2030)	IHC, 1:500; ICC, 1:1000
mouse anti-POU5F1 (OCT3/4)	Santa Cruz Biotechnologies (SC5279)	IHC, 1:50; ICC, 1:100
LIVE/DEAD APC.Cy7(viability)	Invitrogen (L-34975)	FC, 1:1000
mouse anti-CD90 (THY1) PE.Cy7	BD Biosciences (561558)	FC, 1:50
rat anti-CD49f (ITGA6) BV510	BD Biosciences (563271)	FC, 1:50
rabbit anti-Ki67	Epredia (RM-9106-S0)	IHC, 1:500; ICC, 1:500
donkey anti-mouse Alexa fluor 647	Invitrogen (A-31571)	IHC, 1:500; ICC, 1:500
donkey anti-goat Alexa fluor 546	Invitrogen (A-11056)	IHC, 1:500; ICC, 1:500
donkey anti-rabbit Alexa fluor 488	Invitrogen (A-21206)	IHC, 1:500; ICC, 1:500
donkey anti-mouse Alexa fluor 488	Invitrogen (A-21202)	IHC, 1:500; ICC, 1:500
donkey anti-goat Alexa fluor 594	Invitrogen (A-11058)	IHC, 1:500; ICC, 1:500

ICC, immunocytochemistry; IHC, immunohistochemistry; FC, flow cytometry; Cat. No., catalogue number.

Table S2. Total number of DAPI+ cells counted in each biological donor (associated with Table 1).

	donor 1	donor 2	donor 3	Mean DAPI+ cells ± SD
**Shinohara-medium, with EGF, FGF2, GDNF, LIF, estrogen and progesterone**				
**D3**				
**gelatin**	395	1172	809	**792,0 ± 388,8**
**laminin**	792	672	2314	**1259,3 ± 915,3**
**matrigel**	2026	804	448	**2252,3 ± 356,6**
**vitronectin**	1847	2518	2392	**1092,7± 827,7**
**D6**				
**gelatin**	2839	1934	1312	**2028,3 ± 767,9**
**laminin**	2127	1486	5180	**2931,0 ± 1973,9**
**matrigel**	3544	1880	1569	**1405,0 ± 694,3**
**vitronectin**	2147	1297	771	**2331,0 ± 1061,9**
**Zhou-medium (adapted), with RA, BMP4 and ActA**				
**D3**				
**gelatin**	673	1448		**1060,5 ± 548,0**
**laminin**	1348	509		**928,5 ± 593,3**
**matrigel**	1347	1427		**1148,5 ± 7,8**
**vitronectin**	1143	1154		**1387,0 ± 56,6**
**D6**				
**gelatin**	471	1235		**853,0 ± 540,2**
**laminin**	3125	542		**1833,5 ± 1826,5**
**matrigel**	3231	2407		**1955,5 ± 170,4**
**vitronectin**	2076	1835		**2819,0 ± 582,7**
